# A novel puzzle ring replacement technique of a lumen‐apposing metal stent with a double‐pigtail plastic stent to prevent walled‐off necrosis recurrence (with video)

**DOI:** 10.1002/deo2.70020

**Published:** 2024-10-14

**Authors:** Shuntaro Mukai, Atsushi Sofuni, Takayoshi Tsuchiya, Reina Tanaka, Ryosuke Tonozuka, Kenjiro Yamamoto, Yukitoshi Matsunami, Kazumasa Nagai, Hiroyuki Kojima, Hirohito Minami, Kyoko Asano, Takao Itoi

**Affiliations:** ^1^ Department of Gastroenterology and Hepatology Tokyo Medical University Tokyo Japan

**Keywords:** double‐pigtail plastic stent, endoscopic ultrasound‐guided transluminal pancreatic fluid collection drainage, lumen‐apposing metal stent, necrotizing pancreatitis, walled‐off necrosis

## Abstract

Replacing a lumen‐apposing metal stent (LAMS) with a double‐pigtail plastic stent (DPS) after treatment for walled‐off necrosis contributes to the prevention of recurrence. However, the success rate is not very high. To overcome this issue, we devised a novel stent‐replacement technique. In the final treatment procedure, a 7‐F DPS was placed in the lumen of the LAMS. Subsequently, the walled‐off necrosis shrank, and granulation formed over the pigtail portion, which fixed the DPS. The LAMS alone was removed with grasping forceps, leaving the DPS in the lumen of the LAMS (i.e., a puzzle‐ring technique; direct or rotary removal technique). Between August 2021 and August 2023, 18 patients were evaluated for recurrence prevention using this novel technique (median duration of LAMS placement, 37 days). In 17 patients (94.4%), the LAMS was successfully replaced with a 7‐F DPS (direct technique 14, rotary technique 3; median removal procedure time, 3 min). No recurrence was observed during the median observation period of 385 days. Before using this technique (April 2012 to August 2022), the technical success rate of replacement of LAMS with 7‐F DPS was significantly lower (61.8% [42/68, *p* = 0.02]). Recurrence of pancreatic fluid collection occurred in 15.3% (4/26) of the patients who could not undergo replacement with a 7‐F DPS. The novel puzzle ring technique, which improves the success rate of LAMS for DPS replacement, may be useful in reducing recurrence after walled‐off necrosis treatment.

## INTRODUCTION

In recent years, an endoscopic step‐up approach using endoscopic ultrasound‐guided transluminal pancreatic fluid collection drainage (EUS‐PFD) with a lumen‐apposing metal stent (LAMS) has been established for walled‐off necrosis (WON) after necrotizing pancreatitis, with the addition of more invasive endoscopic necrosectomy via the placed LAMS as required.^1^, ^2^ Although a high clinical response rate of this technique has been reported, some concerns have been raised regarding specific LAMS‐related delayed adverse events, including buried stent syndrome due to a high lumen‐apposing force and delayed bleeding due to mechanical impingement of the distal stent flange.^3^ Therefore, if WON resolution is achieved, then the LAMS should be removed within 3–4 weeks after placement.

However, many WON patients have been reported to have pancreatic duct damage, including disconnected pancreatic duct syndrome (DPDS), in which the main pancreatic duct is disrupted, or branch pancreatic duct disruption, causing recurrence of pancreatic fluid collection (PFC) due to continued leakage of pancreatic juice.^4^ Therefore, replacing the LAMS with a double‐pigtail plastic stent (DPS) that can be left in place for a long time contributes to the prevention of PFC recurrence.^5^ However, WON may shrink so quickly that when the LAMS is removed, there is no space for the DPS to be placed, and the success rate of this replacement is not high.

To overcome this issue, we devised a novel stent replacement technique from the LAMS to the DPS, which we call the puzzle ring technique. In the present retrospective study, we evaluated the efficacy of this novel replacement technique.

## TECHNIQUE

### Conventional stent replacement technique

The conventional stent replacement technique was performed between April 2012 and July 2021. Symptomatic WON was treated using an endoscopic step‐up approach using the LAMS with or without an electrocautery‐enhanced delivery system (Hot AXIOS Stent and Delivery System; Boston Scientific; PLUMBER stent; MI Tech). At 3–4 weeks after placement, the LAMS was removed. The flange of the LAMS was grasped using snare forceps and the LAMS was removed through the scope. A 5‐ or 7‐F DPS (Zimmon Biliary Stent Sets; Cook Endoscopy; Through & Pass, Double Pigtail; Gadelius Medical Co., Ltd.) was placed from the fistula in the remaining cavity of the WON. After stent replacement, routine computed tomography or magnetic resonance cholangiopancreatography (MRCP) was performed to check for PFC recurrence. The DPS remained in place for at least one year without replacement to prevent PFC recurrence. When there was no space to place the DPS, only LAMS removal was performed.

### Novel stent replacement technique (puzzle ring technique)

**FIGURE 1:**
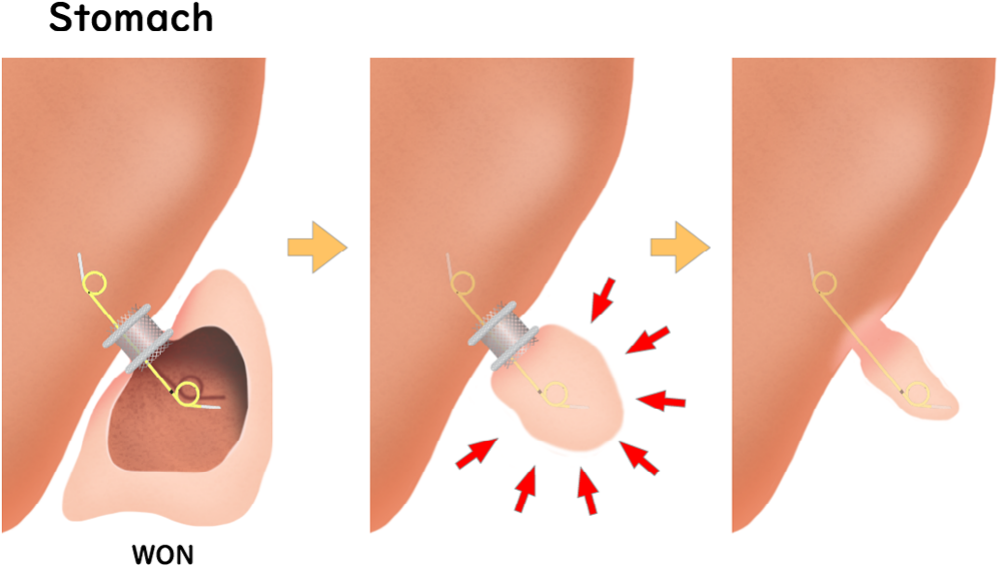
Schema showing the concept of the novel puzzle ring replacement technique.

**FIGURE 2:**
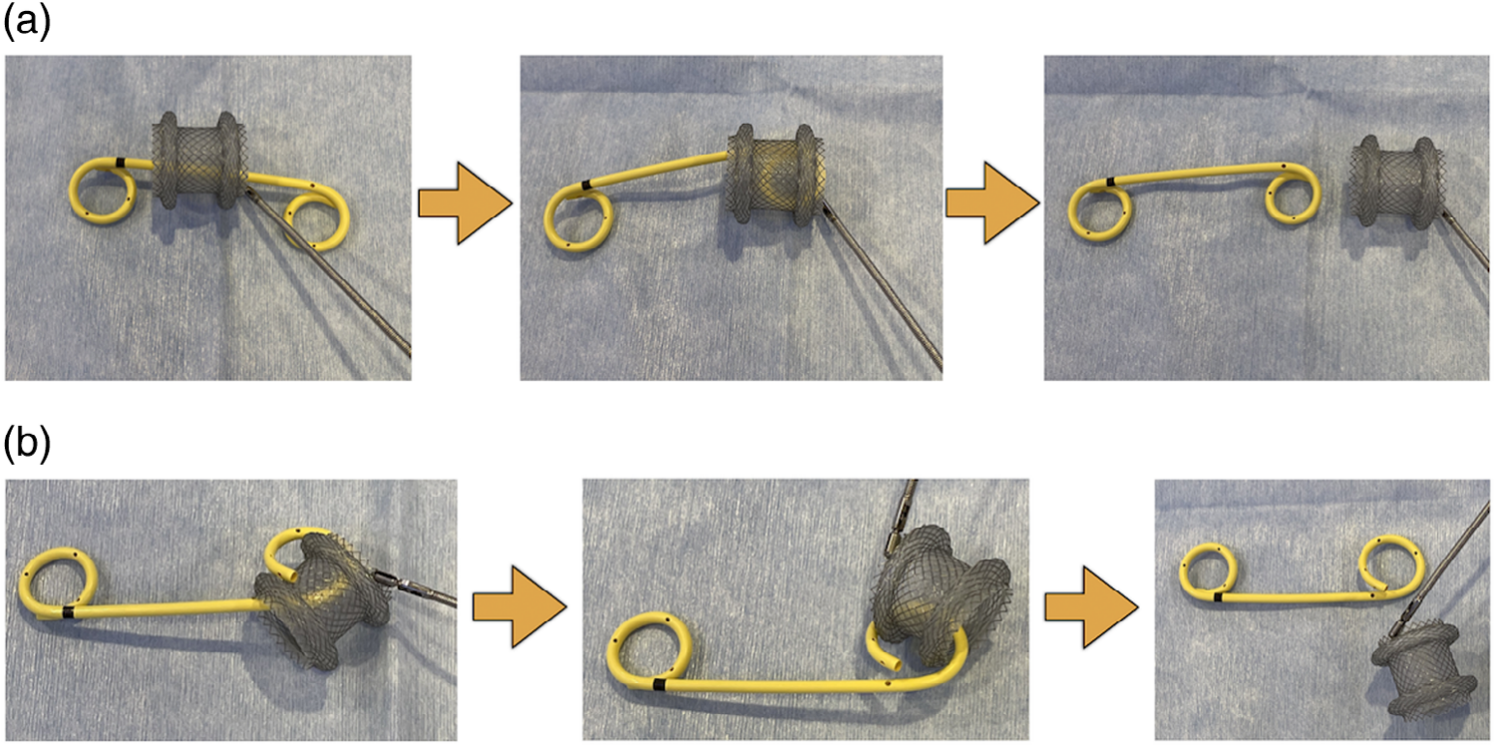
Images of the novel puzzle ring replacement technique. (a) Direct lumen‐apposing metal stent removal technique. (b) Rotary lumen‐apposing metal stent removal technique.

In August 2021, a novel stent replacement technique was performed. Symptomatic WON was treated using an endoscopic step‐up approach with LAMS, as previously described. In the final session of the treatment procedure, 1 DPS with a 15‐mm pigtail part (7‐F diameter, Through & Pass, Double Pigtail; Gadelius Medical Co., Ltd.) was placed from the lumen of the LAMS. After that, the WON shrank, and granulation formed over the pigtail portion on the WON side, which somewhat fixed the DPS placed through the lumen of the LAMS. At 3–4 weeks after placement, the LAMS alone was removed using a therapeutic duodenoscope, leaving the DPS in the lumen of the LAMS (Figure 1). This novel stent replacement technique was named the puzzle ring technique, as it resembles solving a puzzle ring. There are two approaches that may be used for this technique. The first method is the direct removal technique. This approach involves grasping the flange of the LAMS with grasping forceps and passing the pigtail part of the DPS through the stent lumen of the LAMS to remove it directly (Figure 2a; Videos S1 and S2). The other approach is rotary removal. The direct technique was first tried in all cases, and when the endoscopist judged that LAMS removal was difficult with this technique, rotary removal was attempted as an alternative technique. The rotary technique is that the LAMS is removed by skillfully rotating it along the pigtail part of the DPS using grasping forceps (Figure 2b;Videos S3 and Video S4).

### Patients

Between August 2021 and August 2023, 18 patients (14 men and 4 women; median age 67 years old, interquartile range [IQR] 59–75 years old) treated with an endoscopic step‐up approach via LAMS placement for symptomatic WON were evaluated for PFC prevention with this novel technique (LAMS diameter 15 mm in 17 cases and 20 mm in one case; median duration of LAMS placement 37 days, IQR 28–49 days). Patient characteristics and diseases are shown in Table 1. In nine cases of WON, treatment was successfully achieved with EUS‐guided drainage alone, whereas in the other nine, additional endoscopic necrosectomy was required.

**TABLE 1:** Patient characteristics and details of walled‐off necrosis (WON) treatment.

	Patients (*n* = 18)
Age, years, median (IQR)	67 (59–75)
Gender, men/women	14/4
Diameter of LAMS, *n*	
15 mm	17
20 mm	1
Location of LAMS placement, *n*	
Gastric body	17
Gastric antrum	1
Additional necrosectomy	
Necrosectomy via LAMS	9
Drainage only	9
Coaxial DPS within LAMS	
7‐F 4 cm	10
7‐F 7 cm	1
7‐F 10 cm	1
7‐F 12 cm	4
7‐F 15 cm	2
Duration of LAMS placement, days, median (IQR)	37 (28–49)

Abbreviations: IQR, interquartile range; LAMS, lumen‐apposing metal stent; WON, walled‐off necrosis.

Written informed consent for the replacement procedure was obtained from all patients. This retrospective study was approved by our Institutional Review Board (No. T2023‐0159).

### Outcome measurements

We calculated the technical success rate, procedure‐related adverse event rate of the stent replacement procedure, late adverse event rate of long‐term placement of the DPS, and PFC recurrence rate. The technical success of this technique was defined as successful LAMS removal, leaving the 7‐F DPS in the lumen of the LAMS. Cases with spontaneous DPS dislodgement within 1 month were defined as technical failure. Procedure time was defined as the time from the start of LAMS removal until the LAMS was removed from the patient's body. Adverse events were graded according to the severity grading system of the American Society for Gastrointestinal Endoscopy lexicon.

### Comparing the conventional and novel techniques

To further analyze the efficacy of the novel puzzle‐ring technique for the prevention of PFC recurrence, the technical success rate of stent replacement and the PFC recurrence rate were compared with those of the conventional technique. To compare the clinical outcomes accurately, cases that could not be followed up at our hospital for over 3 months were excluded. Thus, the outcomes of 18 patients in the novel technique group were retrospectively compared with those of 65 patients in the conventional technique group (48 men and 17 women; median age, 61 years old, IQR 43–75 years old).

### Statistical analyses

Continuous variables were presented as medians with IQRs. Categorical variables were compared using the chi‐squared or Fisher's exact test. Statistical analyses were performed using the Statistical Package for the Social Sciences software program, version 26 (IBM). A *p*‐value of <0.05 was considered to indicate a statistically significant difference.

## RESULTS

The details of the novel puzzle ring technique for replacing a LAMS with a DPS are shown in Table 2. In 17 patients, the LAMS was successfully replaced with a 7‐F DPS using the puzzle ring technique (technical success rate: 94.4% [17/18]) with no procedure‐related adverse events. In 14, the LAMS was removed using the direct removal technique(technical success rate: 77.8% [14/18]), while in three cases, direct removal was difficult, and the LAMS was instead removed using the rotary removal technique(technical success rate: 100%[3/3]). In one case (technical failure), the 7‐F DPS was removed together with the LAMS when attempting the direct removal technique, and a 5‐F DPS was placed at the fistula site instead. The median procedure time from grasping the LAMS with forceps to its successful removal was 3 (IQR, 2–5) min (the direct removal technique: 2 min [IQR, 1–4], the rotary removal technique: 4 min [IQR, 3–7]). In two cases, a change in the operator to a more advanced physician was required. In 17 successful cases, no PFC recurrence and no late adverse events of long‐term placement of 7‐F DPS occurred during a median observation period of 385 (IQR, 162–610) days.

**TABLE 2:** Details of a novel puzzle ring technique for the replacement of a lumen‐apposing metal stent (LAMS) with a double‐pigtail plastic stent (DPS).

	Patients (*n* = 18)
Overall technical success, *n* (%) Direct removal technique success Rotary removal technique success	17/18 (94.4) 14/18 (77.8) 3/3 (100)
Procedure‐related adverse events, *n* (%)	0/18 (0)
Procedure time, min, median (IQR) Direct removal technique Rotary removal technique	3 (2–5) 2 (1–4) 4 (3–7)
Change of operator, *n* (%)	2/18 (11.1)
Spontaneous DPS dislodgement within 1 month	0/18 (0)
Late adverse events rate of long‐term placement of DPS	0/17 (0)
Recurrence of pancreatic fluid collection	0/17 (0)
Observation period, days, median (IQR)	385 (162–610)

Abbreviations: DPS, double‐pigtail plastic stent; IQR, interquartile range; LAMS, lumen‐apposing metal stent.

A comparison between the conventional technique and the novel puzzle‐ring technique is shown in Table 3. Before using this novel technique, the technical success rate of replacing a LAMS with a 7‐F DPS by the conventional technique was significantly lower (61.8% [42/68, *p* = 0.02]). PFC recurrence occurred in 15.3% (4/26) of patients who could not undergo replacement with a 7‐F DPS and required re‐intervention by EUS‐guided drainage.

**TABLE 3:** Comparing the conventional stent replacement technique with the novel puzzle ring technique.

	Conventional technique (*n* = 68)	Puzzle ring technique (*n* = 18)	*p*‐value
Technical success of 7‐F DPS replacement, *n* (%)	42/68 (61.8)	17/18 (94.4)	0.02
Overall recurrence of pancreatic fluid collection, *n* (%)	4/68 (5.9)	0/18 (0)	0.29
7‐F DPS replacement success case, *n* (%)	0/42 (0)	0/17 (0)	
7‐F DPS replacement failure case, *n* (%)	4/26 (15.4)	0/1 (0)	

Abbreviation: DPS, double‐pigtail plastic stent.

## DISCUSSION

The present study showed that the novel puzzle ring stent replacement technique had a technical success rate of 94.4%, which was significantly higher than that of the conventional technique. Although there was no significant difference in the PFC recurrence rate, the recurrence rate tended to be lower with the novel technique than the conventional technique, owing to the higher stent replacement success rate.

In some cases of WON, the main pancreatic duct disruption occurs due to extensive pancreatic necrosis, resulting in loss of connection between the head‐side and tail‐side pancreatic ducts and prolonged leakage of pancreatic juice into the cavity of the WON. DPDS is reported to occur in 16%–23% of WON cases, and early removal of the stent in patients complicated with DPDS causes recurrence in about 50% of cases.^6^ Therefore, it is necessary to maintain the stent in the WON cavity for a long time. Pawa et al.^5^ reported that the PFC recurrence rate was significantly lower in cases in which the LAMS was replaced with a DPS than without any replacement (5% vs. 37%, *p* = 0.03). However, the success rate of replacing the DPS is not very high (44%), which has been pointed out as an issue. This is because the WON often shrinks at the time of removal which leads to insufficient space for the DPS to be placed. Bang et al.^7^ reported that the success rate of replacement of a LAMS with a DPS was 74%, and PFC recurrence occurred in 25% of patients with DPDS who had unsuccessful DPS replacement. The Orlando protocol recommends that a multi‐gate strategy be adopted for cases of large WON complicated by DPDS.^8^ This strategy involves the placement of a LAMS into one tract in the proximal‐most part of the WON and a DPS into a second tract located more distally towards the pancreatic tail. This strategy can be used to ensure that the LAMS is removed after treatment, while the DPS is left in place, preventing PFC recurrence. Although it is considered a useful strategy, it has the disadvantage that it is not a good indication for WONs <10 cm in size and that it requires two EUS‐guided drainage procedures, which increases the device cost.

The novel puzzle‐ring technique has several advantages as a technique for replacing a LAMS with a DPS. First, the success rate of replacement can be significantly improved. The success rate of the conventional technique was found to be 61.8% in the present study, and using our novel technique improved the success rate to 94.4%. Second, with the conventional technique, even if the DPS could be replaced, it was sometimes spontaneously dislodged early after the replacement due to not being adequately fixed. However, with this technique, the pigtail portion of the DPS on the WON side is firmly fixed by granulation and considered less likely to be dislodged. Third, the presence of a coaxial DPS within the LAMS may reduce the risk of LAMS‐related adverse events. A recent RCT reported that a coaxial DPS in the LAMS reduces the risk of bleeding by preventing LAMS impaction on the adjacent vasculature in the surrounding granulation tissue and reduces the risk of infection by preventing stent occlusion due to necrotic tissue or residual poor drainage areas in the WON cavity.^9^ Fourth, this technique is useful for reducing device costs.

One disadvantage of this technique is that the procedure is complicated. This is especially difficult in cases where the LAMS is placed in the upper part of the gastric body close to the eruption. Actually, of such cases, one case required nearly 10 min for LAMS removal, and in two cases, the physician had to be changed to a more experienced person. In addition, the length of the DPS that is placed in the lumen of the LAMS may affect the difficulty of this technique. The short length of the DPS (4 cm) makes it somewhat more difficult, as the LAMS and the pigtail part are more likely to interfere with each other. Of the three cases in which direct removal was difficult and was converted to rotary removal, two were cases in which a 4 cm DPS was placed. On the other hand, if the DPS is too long, the pigtail part may interfere with the stomach wall, making these procedures difficult. A DPS of 7–10 cm in length may be appropriate for this technique, but the number of cases is small, and further evaluation is required. However, ultimately the procedure was successful in many cases somehow (technical success rate 94.4%). Furthermore, even if this technique fails, the fistula is completely formed, and technical failure will not lead to trouble. It can be converted to the conventional technique. Therefore, this technique should be attempted first. If a DPS with a small pigtail on the gastrointestinal side were to be developed in the future, this technique might become even easier to perform.

Whether or not a LAMS should be replaced with a DPS in all cases is controversial. The degree of pancreatic duct disruption and the presence of DPDS is important in terms of the long‐term outcomes and prevention of recurrence. However, there is no established evaluation method. Bang et al.^10^ reported that the main pancreatic duct was evaluated by EUS observation during an EUS‐guided drainage procedure, and if the main pancreatic duct penetrates the WON cavity and cannot be followed, a high rate of DPDS can be identified, which is useful in selecting the drainage technique and considering stent removal or replacement with a DPS. Although MRCP findings are useful in the evaluation of DPDS and pancreatic duct disruption, the presence of PFC during the index intervention limits the accuracy of MRCP in ascertaining ductal integrity. The usefulness of pancreatic duct evaluation by ERCP and bridging pancreatic duct stenting for DPDS or pancreatic duct disruption has also been reported, but for which cases it is indicated and when it should be performed remain debatable.^11^ Therefore, we attempt to replace LAMS with DPS in all cases, regardless of the presence or absence of DPDS or pancreatic duct disruption. Furthermore, the safety of a long‐term indwelling DPS is unclear. Although rare, long‐term indwelling DPSs can cause late adverse events, such as perforation into the adjacent intestinal tract or pyothorax due to perforation into the thoracic cavity.^12^, ^13^ Therefore, in recent years, indwelling DPSs have been removed, without permanent placement, at our institution. However, there is no evidence supporting the optimal timing, so this issue needs to be resolved in the future.

Several limitations associated with the present study warrant mention. First, it had a single‐center, retrospective design. Multicenter prospective studies are required to validate the efficacy and safety of this novel technique. Second, the sample size was quite small. Further experience and analyses of cases, including improvements in techniques, are warranted. Whether or not this ultimately contributes to a reduction in the PFC recurrence rate also requires a further comparative evaluation in a larger number of cases. Third, the presence of DPDS or pancreatic duct disruption has been reported to be an important factor in recurrence after WON treatment. However, accurate assessment of this point in the WON treatment process is difficult, and this retrospective study cannot show accurate data. This assessment may be an important factor when comparing recurrence rates in further prospective studies of large numbers of cases.

In conclusion, given that no PFC recurrence occurred in this study in patients who successfully underwent replacement of the LAMS with a DPS, replacement of a LAMS with a DPS helps prevent PFC recurrence. Although the novel puzzle ring technique is a somewhat complicated procedure, it can help improve the success rate of LAMS‐to‐DPS replacement and may ultimately reduce PFC recurrence.

## CONFLICT OF INTEREST STATEMENT

Author Shuntaro Mukai has received honoraria for lectures from Gadelius Medical Co. Author Takayoshi Tsuchiya has received honoraria for lectures from Gadelius Medical Co. and Boston Scientific. Author Takao Itoi has received consulting fees from Gadelius Medical Co. and Boston Scientific. Author Takao Itoi is the Editor‐in‐Chief of DEN Open. Author Shuntaro Mukai is an Associate Editor of DEN Open.

## ETHICS STATEMENT

Approval of the research protocol by an Institutional Reviewer Board. This retrospective study was approved by our Institutional Review Board (No. T2023‐0159).

## PATIENT CONSENT STATEMENT

Written informed consent for the replacement procedure was obtained from all patients.

## CLINICAL TRIAL REGISTRATION

N/A

## Supporting information

VIDEO S1 Procedure of direct LAMS removal technique 1.

VIDEO S2 Procedure of direct LAMS removal technique 2.

VIDEO S3 Procedure of rotary LAMS removal technique 1.

VIDEO S4 Procedure of rotary LAMS removal technique 2.
